# *Bacillus velezensis* LW-66: A Broad-Spectrum Biocontrol Agent Against Apple Tree Canker and Other Plant Fungal Diseases

**DOI:** 10.3390/microorganisms14040889

**Published:** 2026-04-16

**Authors:** Dandan Liu, Wei Xiao, Wenwen Li, Shengli Li, Juanli Cheng, Jinshui Lin

**Affiliations:** 1 Shaanxi Key Laboratory of Research and Utilization of Resource Plants on the Loess Plateau, College of Life Sciences, Yan’an University, Yan’an 716000, China; 17829553016@163.com (D.L.); 18792388186@163.com (W.X.); 15129750408@163.com (W.L.); 18181017935@163.com (S.L.); 2 State Key Laboratory for Crop Stress Resistance and High-Efficiency Production, Northwest A&F University, Yangling 712100, China

**Keywords:** *Bacillus velezensis*, apple tree canker, biological control, secondary metabolites

## Abstract

Plant fungal diseases, such as apple tree canker caused by *Valsa mali*, have caused severe losses in agricultural production. Traditional chemical fungicides induce drug resistance in pathogens and cause environmental pollution. Therefore, it is of substantial importance to screen efficient and environmentally friendly bacterial strains as potential biocontrol agents. The tea rhizosphere harbors abundant microbial resources, and previous research has identified microorganisms with antifungal activity existing in this environment. Therefore, in this study, we isolated antagonistic bacteria with broad-spectrum biocontrol potential from tea rhizosphere soil. In this study, a strain with strong antagonistic activity against *V. mali* was isolated from tea rhizosphere soil. Based on morphological characteristics, 16S rRNA gene sequencing, and whole-genome analysis, the isolated strain was identified as *Bacillus velezensis* and designated as LW-66. This strain demonstrated broad-spectrum antifungal activity against various plant pathogenic fungi, including *Valsa mali*, *Fusarium graminearum*, *Bipolaris sorokinianum*, *Alternaria solani*, and *Exserohilum turcicum*. The active extract of *B. velezensis* maintained strong stability across a wide range of temperatures (25–90 °C) and pH values (2–8), with stability decreasing only when the temperature reached 100 °C or pH ≥ 10. In a preventive assay using detached apple branches inoculated with *V. mali*, the control efficacy of LW-66 against apple tree canker reached more than 90%. Additionally, in a therapeutic assay using *V. mali*-infected potted apple seedlings, the LW-66 bone-glue bacterial agent achieved a survival rate of up to 90%. Whole-genome analysis revealed that the genome of LW-66 contains 13 predicted secondary metabolite biosynthetic gene clusters, seven of which showed high homology (≥92% similarity) with known antimicrobial gene clusters, including surfactin, bacillaene, macrolactin H, fengycin, difficidin, bacillibactin, and bacilysin. These gene clusters may be connected to the broad-spectrum antifungal activity of *B. velezensis*, as well as its ability to disrupt hyphal morphology. The volatile organic compounds produced by LW-66 inhibited *V. mali* growth by 91.70%. Collectively, these findings demonstrate that *B. velezensis* LW-66 has a wide antimicrobial range and strong antagonistic effects against multiple plant pathogenic fungi. Therefore, *B. velezensis* shows promise as a biocontrol agent for managing fungal diseases in plants, providing a basis for developing LW-66-derived biocontrol products aimed at controlling diseases such as apple tree canker.

## 1. Introduction

Fungi are one of the primary causes of plant diseases [1,2], resulting in substantial economic losses in agricultural production. Various common plant diseases, including powdery mildew, leaf spot, canker, wilt, and rust, are caused by fungi [3,4,5,6,7,8]. Currently, chemical fungicides are the main method used to control plant fungal diseases, with agents such as aminobenzimidazole and phthalimide being widely used [9]. However, long-term application of such chemical agents not only readily induces resistance in pathogenic fungi but also gives rise to a series of potential risks threatening food security, including environmental pollution and pesticide residues in agricultural products [10,11,12]. Therefore, it is critical to develop efficient and safe methods for the control and prevention of plant fungal diseases.

Biological control, due to its prominent advantages, such as being environmentally friendly, highly sustainable, non-toxic, pollution-free, low risk of inducing pathogen resistance, and high safety, has been recognized as one of the safest and most effective approaches for controlling various plant fungal diseases through utilizing microbial resources [13,14]. In recent years, increasing research has focused on developing microbial-based biocontrol products targeting fungal pathogens in plants. Among diverse biocontrol resources, the bacterial genus *Bacillus* has been widely developed as an agricultural biocontrol agent due to its outstanding pathogen inhibition capabilities [15]. Notably, *Bacillus velezensis*, an emerging class of antagonistic microorganisms, can generate a variety of secondary metabolites, including lipopeptide antibiotics, polyketides, antimicrobial proteins, and other bioactive substances, thereby achieving broad-spectrum inhibition against plant pathogens. *B. velezensis* is currently widely deployed as a biocontrol agent for the management of various plant diseases [16,17]. For example, an endophytic *B. velezensis* strain isolated from maize seeds was shown not only to exhibit remarkable inhibitory effects against various maize pathogenic fungi but also to harbor an abundance of biocontrol-related functional genes [18]. *B. velezensis* OEE1, which has been isolated from olive tree internal tissues, demonstrates potent antifungal activity against Verticillium dahliae, the pathogen responsible for olive verticillium wilt, while also enhancing olive tree growth [19]. Similarly, *B. velezensis* YB-130, obtained from diseased wheat spike tissues, exhibits broad antifungal properties through the inhibition of the spore germination and mycelial development of fungi such as Fusarium graminearum [20]. *B. velezensis* strain YYC was reported not only to promote the growth of tomato plants but also to significantly reduce the incidence of tomato bacterial wilt [21]. In summary, *B. velezensis*, as a novel biocontrol resource possessing both antifungal and growth-promoting activities, exhibits comprehensive research and application prospects in the field of green prevention and control of plant diseases.

China accounts for about half of the world’s apple production and cultivation area [22]. However, diseases severely restrict the high-quality development of China’s apple industry. Among them, apple Valsa canker caused by *V. mali* is a devastating disease, characterized by its wide distribution, serious damage, and difficult control, making it known as the “cancer” of apple trees [23,24,25,26]. *V. mali* causes extensive branch dieback, tree decline, and even orchard failure, leading to substantial economic losses. A national survey reported that the average incidence of Valsa canker in China reached 52.7%, with 30–80% in seriously affected orchards and even 100% individual severely infected orchards. This disease causes billions of yuan in direct economic losses annually and poses a severe threat to the sustainable development of the apple industry [27,28,29].

Currently, several studies have been reported on the utilization of biological control methods to prevent and manage apple tree canker. For example, *Bacillus amyloliquefaciens* GB1 was isolated from senescent cucumber stems, and this strain exhibited strong antagonistic activity against *V. mali*, significantly inhibiting its conidial germination and mycelial growth and thereby effectively controlling the occurrence of the disease [30]. *Bacillus amyloliquefaciens* H12 was isolated from the soil, which not only directly induced the death of *V. mali* hyphae but also increased the immune response of apple plants and modulated the structure of the phyllosphere microbial community. With both antifungal and growth-promoting properties, *B. amyloliquefaciens* displayed promising application potential [31]. Although biocontrol has demonstrated potential in laboratory studies, the large-scale field application of microbial biocontrol agents still faces two crucial bottlenecks: (1) the discovered efficient biocontrol strain resources are relatively limited, restricting the development of bacterial biocontrol agents; and (2) exogenous biocontrol strains often struggle to adapt to the complex field environments and fail to colonize stably on plant surfaces or internally, resulting in inconsistent control efficacy [32]. However, few studies have characterized *Bacillus velezensis* strains isolated from tea rhizosphere soils, and their potential for controlling apple canker remains unexplored. Accordingly, the present study aims to systematically evaluate the broad-spectrum antifungal activity, stability of bioactive metabolites, and in vivo control efficacy of strain LW-66 against *V. mali* in apple trees. These results offer a theoretical foundation for developing LW-66-based biocontrol agents and support environmentally friendly strategies for controlling fungal diseases such as apple Valsa canker.

## 2. Materials and Methods

### 2.1. Samples and Test Strains

Soil samples for bacterial isolation were collected from the rhizosphere of tea plants in Fenghuang Tea Plantation, Jiangjiaping Village, Pingli County, Ankang City, Shaanxi Province, China. A total of three tea rhizosphere soil samples were collected at a depth of 10–15 cm, and each sample was processed individually. Five pathogenic fungi were utilized in this study: *V. mali*, *F. graminearum*, *A. solani*, *B. sorokinianum*, and *E. turcicum*. All pathogens employed in the present study were obtained from the Laboratory of Pathogenic Microorganisms, College of Life Sciences, Yan’an University.

### 2.2. Isolation and Screening of Bacterial Strains

The methodology of Liangsheng Xu et al. [33] was followed, with suitable modifications. In brief, 1 g of each soil sample was suspended in 100 mL of sterile distilled water and incubated at 37 °C with shaking at 200 rpm for 30 min. Subsequently, 1 mL of this suspension was serially diluted (10-fold) up to 10^−6^. For each dilution, 0.1 mL was spread onto Luria–Bertani (LB) agar plates [34], which were then incubated at 37 °C. Single colonies were inoculated onto sterile LB agar medium until pure cultures were obtained. *V. mali* was employed as the indicator strain, and the optimal antagonistic strain was screened utilizing the plate confrontation method [35].

### 2.3. Morphological Characterization

Strain LW-66 was inoculated onto LB, Tryptone Soya Broth (TSB) [36], and Tryptone-Yeast Extract Medium (TY) [37] solid media and incubated at 37 °C for 24 h. Colony morphology was observed. Gram staining was conducted, and morphological characteristics were observed utilizing light microscopy (SOPTOP Optoelectronics Co., Ltd., Ningbo, China, EX33) and scanning electron microscopy (JEOL Ltd., Tokyo, Japan, JSM-7800F). Physiological and biochemical tests were performed according to the Common Bacterial Identification Manual and Bergey’s Manual of Systematics of Archaea and Bacteria [38,39]. Tests included utilization of carbon sources (fructose, maltose, sucrose, glucose, and lactose), V-P test, methyl red test, oxidase test, amylase activity, protease activity, catalase test, and growth in LB liquid medium supplemented with 0%, 3%, 5%, 7%, 10%, and 15% NaCl to assess the salt requirement and tolerance of the microorganism.

### 2.4. Molecular Identification

The molecular identification of strain LW-66 was performed via 16S rRNA gene sequencing. PCR amplification was carried out with the universal bacterial primers 27F (5′-AGAGTTTGATCCTGGCTCAG-3′) and 1492R (5′-GGTTACCTTGTTACGACTT-3′). The 50-μL reaction system was prepared as follows: 1 μL TransTaq-T DNA Polymerase, 2 μL each of forward and reverse primers, 5 μL genomic DNA template, 4 μL dNTP mix (2.5 mmol/L), 5 μL 10× TransTaq T Buffer, and 31 μL sterile ddH_2_O. Thermal cycling conditions were as follows: initial denaturation at 95 °C for 5 min; 31 cycles of 95 °C for 50 s, 55 °C for 1 min, and 72 °C for 90 s; and a final extension at 72 °C for 10 min. Amplified products were verified by 1% agarose gel electrophoresis. The purified ~1500 bp PCR fragment was ligated into the pMD19-T vector, followed by transformation into *Escherichia coli* TG1 competent cells. Positive transformants were screened and sent to Sangon Biotech (Shanghai) Co., Ltd., Shanghai, China. for Sanger sequencing. The obtained 16S rRNA sequence was aligned against reference sequences in the EzTaxon database (http://eztaxon-e.ezbiocloud.net, accessed on 13 January 2026). A maximum-likelihood phylogenetic tree was constructed using BioEdit v7.2.5 and MEGA X v10.2.6 64-bit software [40] to confirm the taxonomic position of strain LW-66.

### 2.5. Determination of the Antimicrobial Spectrum of Strain LW-66

The plate confrontation culture method was utilized to determine the antimicrobial activity of LW-66 [41,42]. Strain LW-66 was inoculated on LB solid medium and incubated at 37 °C for 24 h. Each plant pathogenic fungus was cultured on potato dextrose agar (PDA) [43] for 4–6 d. A mycelial plug (8 mm, mycelium-side down) of each pathogen was placed at the plate center, and strain LW-66 was inoculated at four points 25 mm from the center. Plates were incubated at 25 °C for 4–6 days until the control colony occupied approximately three-quarters of the plate, after which the inhibition zone diameter was measured via the cross-crossover method [44], and the inhibition rate was calculated based on Equation (1). All treatments were performed in triplicate, and the mean value was used to assess the antimicrobial activity of strain LW-66.

(1) I = [(r1 − r2)/(r1 − r3)] × 100%,

where r1 represents the fungal colony diameter in the control group, r2 represents the colony diameter of pathogenic fungi in the presence of bacteria, and r3 refers to the diameter of the pathogenic fungi plug.

### 2.6. Fermentation Culture of Strain LW-66 and Preparation of Active Extracts

Fermentation of strain LW-66 was carried out according to a previously reported protocol [14]. Additionally, strain LW-66 was grown in liquid LB medium at 37 °C with shaking at 200 rpm for 24 h to establish a seed culture. The seed culture was then transferred to TY liquid medium at a ratio of 1:100 and incubated at 37 °C with shaking at 200 rpm for 3 d to obtain fermentation broth. The fermentation broth was centrifuged to separate the supernatant and cells. The supernatant was then filtered through a 0.22-µm sterile membrane filter, extracted with an equal volume of ethyl acetate, and concentrated utilizing a rotary evaporator to obtain the supernatant extract. The cells were soaked in methanol for 1 d, and the methanol extract was collected via centrifugation. The pellet was then soaked in methanol once again and extracted ultrasonically. This procedure was repeated three times until the methanol extract was colorless. The combined methanol extracts were concentrated to obtain the intracellular extract. The supernatant extract and intracellular extract were combined to yield the active extract of the fermentation broth.

### 2.7. Stability Test of the Active Extract of Strain LW-66

The stability experiment was conducted according to an earlier report [45]. An 8-mm mycelial disc of *V. mali* was placed in the center of a PDA plate. Four uniform wells (8 mm in diameter) were created at a distance of 2.5 cm from the fungal disc using a sterile punch. The extract was re-dissolved in methanol to a final concentration of about 38.5 mg/mL, and then subjected to heat treatment at 25 °C, 50 °C, 60 °C, and 100 °C for 30 min. The pH value was adjusted to 2, 4, 6, 8, 10, and 12 with 1 mol/L HCl or 1 mol/L NaOH, which were also used as blank controls. A 200 μL volume of each treated sample (approximately 38.5 mg/mL) was added to the corresponding wells. All plates were incubated at 25 °C for 4–6 days with each treatment performed in triplicate, and inhibitory activity was assessed once the control colony covered approximately three-quarters of the plate.

### 2.8. Inhibitory Effect of Volatile Organic Compounds from Strain LW-66 Against V. mali

We used the method described by Weiqiang Lai et al. with modifications to determine the inhibitory effect of LW-66-produced volatile organic compounds on *V. mali* [46]. An 8-mm mycelial plug of *V. mali* was placed at the center of a PDA plate, and strain LW-66 was inoculated on an LB plate. The two plates were then sealed face-to-face. A plate without LW-66 served as the control. After incubation at 25 °C for 4–6 d until the control colony occupied approximately three-quarters of the plate, at which point the inhibitory activity was evaluated. The inhibitory effect was observed, and the inhibition rate was calculated using the same formula as in Section 2.4 [44]. All experiments were performed in triplicate.

### 2.9. Inhibitory Effect of a Cell-Free Filtrate of Strain LW-66 Against V. mali

A cell-free filtrate of strain LW-66 was prepared as described earlier [47]. Briefly, LW-66 was inoculated into liquid LB medium and cultured at 37 °C with shaking at 200 rpm for 24 h. The seed culture was then transferred to TY medium at a 1% inoculation ratio and incubated under the same conditions for 3 days. The culture broth was centrifuged at 8000 rpm for 10 min at 25 °C, and the resulting supernatant was filtered through a 0.22-μm sterile syringe filter to obtain the cell-free filtrate. The filtrate was incorporated into PDA medium at final concentrations of 1%, 2%, 4%, 8%, and 16%, with PDA medium without filtrate set as the untreated control. An 8-mm mycelial plug of *V. mali* was placed at the center of each plate. Each treatment was performed in triplicate, with two independent biological replicates. Plates were incubated at 25 °C until the control colony occupied approximately three-quarters of the plate, at which point the inhibitory activity was evaluated.

### 2.10. Effect of Strain LW-66 on the Mycelial Morphology of V. mali

The effect of strain LW-66 on the mycelial morphology of *V. mali* was evaluated using the plate confrontation method. Strain LW-66 and an 8-mm mycelial plug of *V. mali* were inoculated on a PDA plate. Mycelia of *V. mali* within the inhibition zone were harvested and observed under an inverted fluorescence microscope (Nikon Corporation, Tokyo, Japan, Eclipse Ti2). Normal mycelia from the non-antagonistic region were utilized as the control. Morphological alterations of the mycelia of *V. mali* were observed and recorded.

### 2.11. Preventive Effect of Strain LW-66 on V. mali Infection in Detached Apple Branches

The preventive effect of strain LW-66 on detached apple branches was assessed utilizing the wound-inoculation method [48]. Perennial Fuji apple branches (12–18 mm diameter) were cut into 25-cm segments, washed with tap water, disinfected with 75% ethanol for 10 min, rinsed three times with sterile water, and then sealed with wax at both ends. Each branch was wounded with a heated iron nail cap (8 mm diameter). LW-66 cells or active extract (undiluted,1/2, 1/5, 1/10, 1/20, 1/50, and 1/100 dilutions) was applied to the wound. After air-drying, an 8-mm *V. mali* plug was inoculated. Each branch contained one inoculation site, and five branches were used per treatment. Sterile water was applied as the control. After incubation at 25 °C under high humidity for 7 d, lesion length was measured using the cross-crossover method, and the lesion area was calculated to determine the inhibitory effect.

(2) S = (1/4)π × d_1_ × d_2_, P = [(S_1_ − S_2_)/S_1_] × 100%.

where S represents the lesion area; d1 and d2 denote the long and short diameters of the lesion, respectively; and S1 and S2 indicate the colony areas of the pathogenic fungus in the control group and after treatment with the antagonistic strain, respectively.

### 2.12. Control Efficacy of a Bone Glue-Based Agent Containing Strain LW-66 on V. mali Infection

The control efficacy of strain LW-66 against *V. mali* in potted apple trees was determined employing the wound-inoculation method [48]. A bone glue-based agent containing strain LW-66 as the active ingredient was prepared according to previous reports [49]. To prepare the bone glue-based agent, we soaked 333.3 g of natural bone glue in 333.3 mL of water overnight. Then, the solution was melted in a 70 °C water bath with stirring, cooled to 30 °C, and mixed with 166.7 mL of glycerol and 3.33 g of CaCO_3_ to form the base. Then, 500 mL of strain LW-66 fermentation broth was added and mixed thoroughly to concoct the bone glue agent, which was stored at room temperature. Three-year-old Fuji apple trees were transplanted into pots and cultivated until new leaves emerged before use in this model.An 8-mm *V. mali* plug was inoculated onto a wounded trunk and fixed with plastic wrap. Trees were covered with transparent plastic bags (60 cm × 90 cm) and incubated under natural light at room temperature with daily misting to maintain high humidity. After successful infection and development of typical canker lesions, the epidermis was scraped off. Trees were divided into three groups: Group 1: no treatment (blank control); Group 2: base only (matrix control); and Group 3: strain LW-66 bone glue agent. The agent was applied every 10 d, and the control efficacy was observed and recorded after 30 d.

### 2.13. Genome Sequencing of Bacillus *sp*. LW-66

#### 2.13.1. Genome Sequencing and Assembly of *Bacillus* sp. LW-66

Strain LW-66 was inoculated on LB medium and incubated at 37 °C for 24 h. A single colony was then inoculated into LB liquid medium and incubated at 37 °C with shaking at 220 rpm for 12 h. The cells were harvested by centrifugation at 6000 rpm for 5 min. Whole-genome sequencing was conducted by Novogene Co., Ltd. (Tianjin, China). SMRT Bell libraries were constructed using the PacBio platform, sequencing was performed on the PacBio Revio system, and genome assembly was performed using Hifiasm software (version 0.14.2-r315).

#### 2.13.2. Gene Prediction and Annotation

We used GeneMarkS (v4.17) to predict protein-coding genes. RepeatMasker (open-4.0.5) and tRNAscan-SE (v1.3.1) were applied to detect dispersed repetitive sequences and tRNAs, respectively. Functional annotation was conducted using DIAMOND v2.1.8 searches against the NR, GO, KEGG, and COG databases and the Swiss-Prot databases, and the best hit with the highest score was retained for each gene. Orthologous gene clusters were identified using OrthoFinder v2.5.4 among all selected species. Single-copy orthologous genes shared with strain LW-66 were extracted for phylogenetic tree construction. These single-copy orthologous genes were aligned and concatenated using MAFFT v7.490. A phylogenetic tree was constructed using RAxML v8.2.12, with subsequent visualization and refinement performed utilizing the Interactive Tree Of Life (iTOL) online tool (https://itol.embl.de/, accessed on 13 January 2026).

#### 2.13.3. Prediction of Secondary Metabolite Biosynthetic Gene Clusters of Strain LW-66

To identify secondary metabolite biosynthetic gene clusters in *B. velezensis* LW-66, we submitted its genome to the antiSMASH web server (http://antismash.secondarymetabolites.org/, accessed on 13 January 2026) for analysis, and we looked for matches to known biosynthetic gene clusters [49].

### 2.14. Statistical Analysis

Each assay was performed with at least three parallel replicates, and all experiments were independently conducted twice as biological replicates. All results are given as mean ± SD. Statistical tests were performed with IBM SPSS 26.0 (IBM Corp., Armonk, NY, USA), including one-way analysis of variance (ANOVA) followed by Dunnett’s test for pairwise comparisons. A *p* value < 0.05 was considered statistically significant, and different lowercase letters in the tables indicated significant differences. Adobe Illustrator 2020 was used to plot and edit all figures.

## 3. Results

### 3.1. Isolation of Strain LW-66

A total of 15 bacterial strains were isolated from the soil samples. Strain LW-66 had the highest activity against *V. mali*, with an inhibition rate of 90.30 ± 0.37% (Supplementary Table S1). Accordingly, this strain was selected for further characterization

### 3.2. Culture and Morphological Observation of Strain LW-66

**Figure 1:**
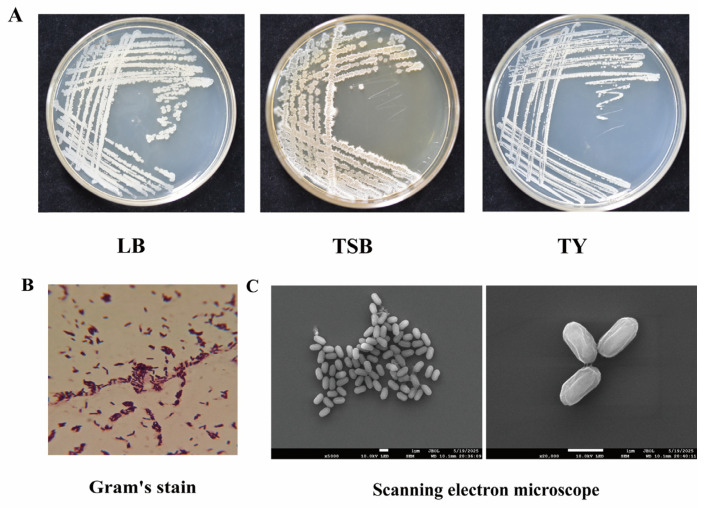
Morphological features of strain LW-66. (**A**) Colony morphology of LW-66 on LB, TSB, and TY media. (**B**) Gram staining of LW-66 observed under light microscopy. (**C**) Morphological characteristics of LW-66 under an electron microscope.

Strain LW-66 was inoculated into LB, TSB, and TY media for culture. The colony phenotypes of this strain on the three types of agar plates were consistent: all appeared milky white, with an irregularly round colony morphology, rough and dry surfaces, dense and opaque textures, and undulate or serrate margins (Figure 1A). Gram staining of LW-66 followed by optical microscopy revealed that the cells were purple, indicating that it was a Gram-positive bacterium (Figure 1B). Scanning electron microscopy showed that strain LW-66 is a short rod-shaped bacterium (Figure 1C).

### 3.3. Analysis of Biological Characteristics of Strain LW-66

The amylase and protease activities of strain LW-66 were evaluated utilizing the plate transparent zone method. After inoculation on starch hydrolysis plates and incubation at 37 °C for 24–48 h, obvious transparent hydrolysis zones appeared around the colonies, indicating that LW-66 can secrete amylase and hydrolyze starch (Figure S1A). After inoculation on protease detection plates and incubation under the same conditions, transparent zones also appeared around the colonies, signifying that LW-66 can secrete protease and hydrolyze proteins (Figure S1B). Other physiological and biochemical characteristics, as well as the salt tolerance of strain LW-66, were assessed. The results demonstrated that strain LW-66 was positive for the methyl red test, oxidase test, catalase test, glucose fermentation, sucrose fermentation, and fructose fermentation, whereas it was negative for the Voges-Proskauer (V-P) test, lactose fermentation, and maltose fermentation. Moreover, LW-66 was able to grow under NaCl concentrations of 3%, 5%, 7%, and 10%, exhibiting broad-spectrum salt tolerance (Table 1).

### 3.4. Molecular Identification Analysis

**Figure 2:**
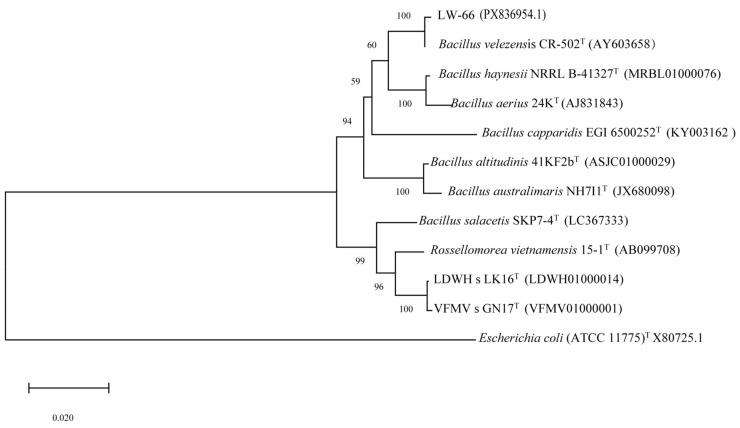
Phylogenetic tree of strain LW-66 constructed based on 16S rRNA gene sequences. The numbers in the parentheses are GenBank or National Microbiology Data Center accession numbers. Values above the branches are parsimony bootstrap. The value on the scale bar represents one nucleotide substitution per site.

The 16S rRNA gene sequence of strain LW-66 was sequenced by Sangon Biotech (Shanghai) Co., Ltd., Shanghai, China, yielding a product length of 1518 bp (GenBank: PX836954.1). Sequence similarity search and alignment were conducted using EzTaxon, which showed that this strain shares high similarity with strains of the genus *Bacillus*, indicating that strain LW-66 is a member of the genus *Bacillus*. A maximum-likelihood phylogenetic tree was constructed using BioEdit and MEGA X 64-bit software. Phylogenetic analysis indicated that strain LW-66 clustered with *B. velezensis* CR-502 on a single branch with strong bootstrap support, with the highest sequence homology between the two strains reaching 99.93% (Figure 2), signifying their close phylogenetic relationship. Therefore, based on morphological and cultural characteristics, strain LW-66 was identified as a member of the genus *Bacillus* and named *Bacillus* sp. LW-66.

### 3.5. Determination of the Inhibition Spectrum of Bacillus *sp*. LW-66

**Figure 3:**
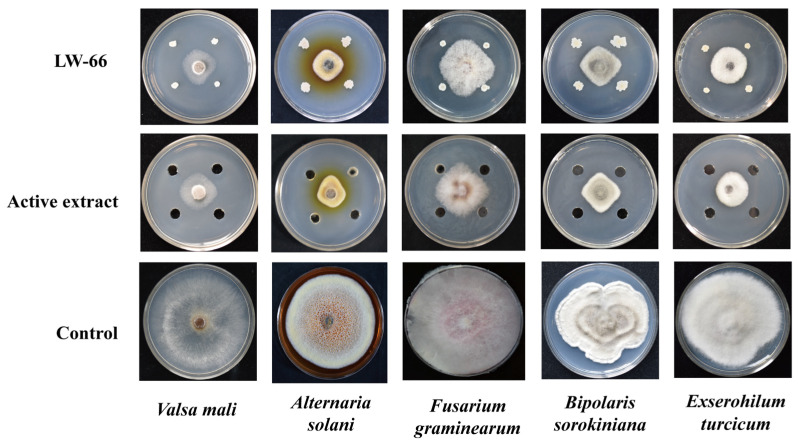
Inhibitory effect of bacterial strain *Bacillus* sp. LW-66 and its active extract on five plant pathogenic fungi. The assay was conducted on PDA medium. Plates were incubated at 25 °C for 4–6 d until the control colony occupied approximately three-quarters of the plate, at which point the inhibitory activity was evaluated. Top row: antagonistic activity of LW-66 cells; middle row: antagonistic activity of LW-66 active extract (final concentration: approximately 38.5 mg/mL); and bottom row: pathogenic fungi cultured alone as the control. The tested pathogens were *V. mali*, *A. solani*, *F. graminearum*, *B. sorokiniana*, and *E. turcicu*.

The antagonistic activities of the bacterial strain *Bacillus* sp. LW-66 and its active extract were evaluated against five plant pathogenic fungi via the plate confrontation assay. We found that both *Bacillus* sp. LW-66 and its intracellular extracts displayed substantial inhibitory effects on all five pathogenic fungi (Figure 3). Among these, the highest inhibition rates were observed against *V. mali*, reaching 90.19 ± 0.37% and 91.68 ± 1.69% for *Bacillus* sp. LW-66 and its intracellular extracts, respectively. The lowest inhibition rates were observed against *F. graminearum*, with values of 69.67 ± 0.71% and 74.80 ± 7.09% (Table S2).

### 3.6. Antifungal Activity of Volatile Substances Produced by Bacillus *sp*. LW-66

**Figure 4:**
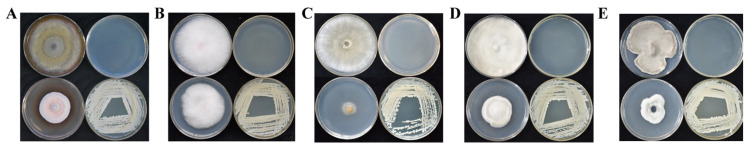
Effect of the volatile substances of *Bacillus* sp. LW-66 on the growth of five plant pathogenic fungi. The assay for the effect of volatile substances was performed on PDA medium. All plates were cultured at 25 °C for 4–6 d until the control colony occupied approximately three-quarters of the plate, at which point the inhibitory activity was evaluated. (**A**): *A. solani*; (**B**): *F. graminearum*; (**C**): *V. mali*; (**D**): *E. turcicum*; and (**E**): *B. sorokinianum*.

In the plate confrontation assay, the volatile substances synthesized by *Bacillus* sp. LW-66 exhibited inhibitory effects on the growth of all five tested plant pathogenic fungi (Figure 4). Among them, the inhibitory effect against *V. mali* was the strongest, with an inhibition rate of 91.70 ± 1.03% (Table S3).

### 3.7. Effect of Cell-Free Filtrate of Bacillus *sp*. LW-66 on the Growth of V. mali

**Figure 5:**
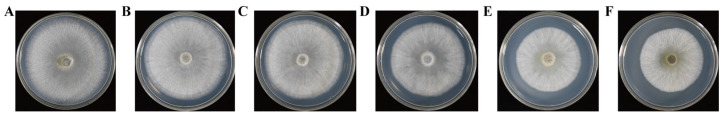
Effects of bacteria-free filtrate from *Bacillus* sp. LW-66 on the growth of *V. mali*. The assay was performed on PDA medium. All plates were cultured at 25 °C for 4–6 d until the control colony occupied approximately three-quarters of the plate, at which point the inhibitory activity was evaluated. (**A**): 0%; (**B**): 1%; (**C**): 2%; (**D**): 4%; (**E**): 8%; and (**F**): 16%.

As shown in Figure 5 and Table 2, cell-free filtrates of *Bacillus* sp. LW-66 exhibited dose-dependent inhibitory activity against the mycelial growth of *V. mali*. The inhibitory effect was significantly enhanced with increasing filtrate concentration, with the 16% cell-free filtrate showing the strongest antagonistic activity against *V. mali* among all tested concentrations.

### 3.8. Stability of the Antifungal Activity of Active Extracts from Bacillus *sp*. LW-66 After Heat and pH Treatments

In the field of agricultural biocontrol, the practical application efficacy of biological agents is intimately associated with their stability. Excellent environmental stability is a core prerequisite for the field promotion of biocontrol agents. Based on this requirement, this study systematically analyzed the environmental stability of the active extracts from *Bacillus* sp. LW-66, and the relevant results are summarized in Table 3 and Table 4. Following treatment across a gradient of temperatures, the active extracts of this strain maintained antagonistic activity under most temperature conditions. A significant decrease in antifungal activity was observed only after treatment at 100 °C. In the pH stability tests, the antagonistic activity of the intracellular extracts was substantially reduced when the environmental pH was ≥10, whereas the antifungal activity remained stable across the other pH ranges. Taken together, these results indicate that the intracellular active substances of *Bacillus* sp. LW-66 possesses suitable tolerance to fluctuations in temperature and pH.

### 3.9. Effect of Bacillus *sp*. LW-66 on the Mycelial Morphology of V. mali

**Figure 6:**
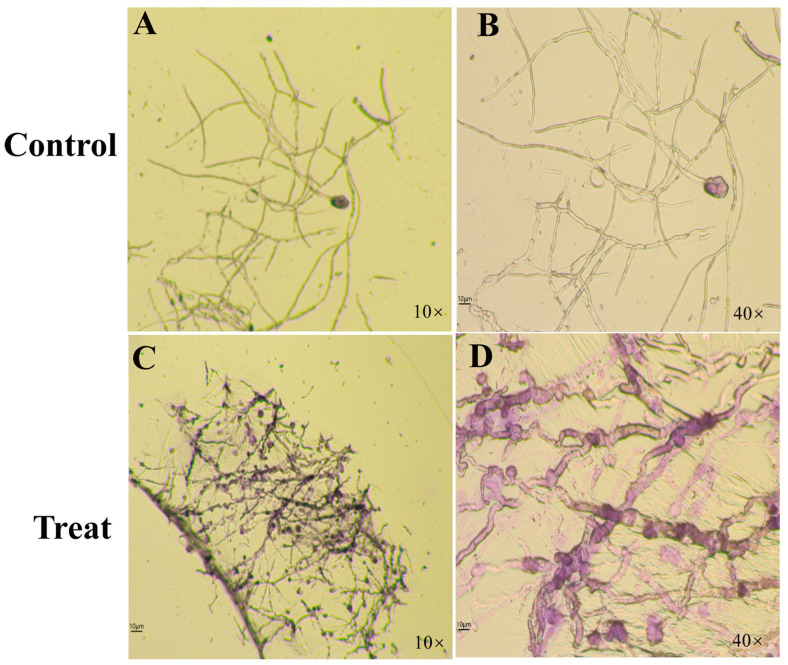
Effects of *Bacillus* sp. LW-66 on the mycelial morphology of *V. mali* during confrontation culture. (**A**,**B**): Mycelial morphology of *V. mali* in the non-confronting area. (**C**,**D**): Mycelial morphology of *V. mali* in the confronting area.

Previous studies have shown that *Bacillus* sp. LW-66 displays broad-spectrum inhibitory activity against various plant pathogenic fungi. However, whether the antifungal effects of LW-66 directly affect the mycelia of these plant pathogens is not clear. Therefore, we used a dual-culture system with *Bacillus* sp. LW-66 and *V. mali* to observe the effect of LW-66 on the mycelial morphology of the plant pathogen *V. mali*. Specifically, mycelia at the edge of the inhibition zone affected by antagonism were selected for microscopic observation, with normally growing mycelia without antagonism as the control. As shown in Figure 6, treatment with *Bacillus* sp. LW-66 induced significant morphological alterations in *V. mali* mycelia. Compared with the normal growth of control hyphae (Figure 6A,B), the antagonized mycelia were characterized by increased thickness, disrupted cell structures, and a markedly darker hyphal pigmentation (Figure 6C,D). These results indicate that the antagonistic effect of *Bacillus* sp. LW-66 alters the mycelial morphology of *V. mali*.

### 3.10. Control Efficacy of Bacillus *sp*. LW-66 Against V. mali

#### 3.10.1. Preventive Activity of *Bacillus* sp. LW-66 in a Detached Apple Branch Assay Against *V. mali*

**Figure 7:**
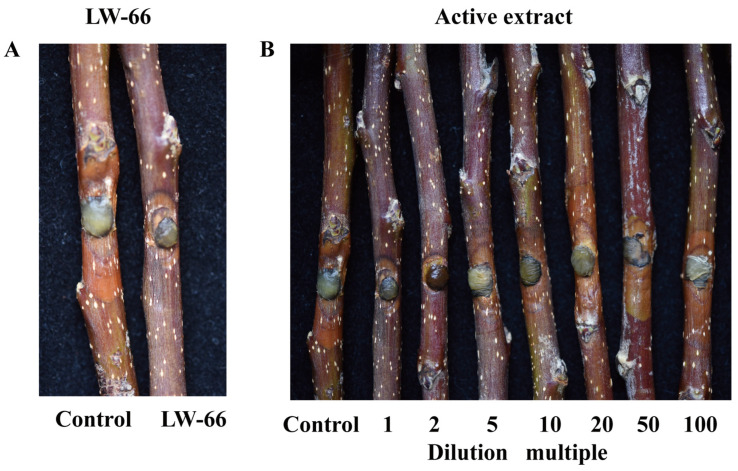
Effect of *Bacillus* sp. LW-66 on the prevention of *V. mali* infection in apple tree branches. (**A**): Apple tree branches were pretreated with or without *Bacillus* sp. LW-66. (**B**): Apple tree branches were pretreated with intracellular extracts of *Bacillus* sp. LW-66 at various concentrations.

To evaluate the in vivo biocontrol potential of *Bacillus* sp. LW-66 against fungal phytopathogens, we initially tested its protective efficacy against *Bacillus* sp. LW-66 in an isolated apple tree branch infection model with *V. mali*. The results are shown in Figure 7, Table 5 and Table 6. Pre-coating with bacterial strain *Bacillus* sp. LW-66 at a concentration of 1 × 10^9^ CFU/mL effectively prevented *V. mali* infection in detached apple branches, thereby significantly inhibiting the expansion of canker lesions, with a preventive efficacy of 91.29 ± 2.36% (Figure 7A and Table 5). Similarly, pre-treatment with active extracts of *Bacillus* sp. LW-66 (initial stock concentration: approximately 38.5 mg/mL) also protected detached apple branches from *V. mali* infection and suppressed lesion extension. Specifically, the undiluted intracellular active extract, as well as the 2-fold and 5-fold dilutions, exhibited potent preventive efficacy against *V. mali* on detached branches, achieving inhibition rates of 92.21%± 1.96%, 91.27 ± 2.27%, and 86.50 ± 3.58%, respectively. Even at a 20-fold dilution, the preventive effect remained above 80% (Figure 7B and Table 6), indicating the high potency of the antifungal metabolites synthesized by LW-66.

#### 3.10.2. Control Efficacy of *Bacillus* sp. LW-66 in *V. mali*-Infected Potted Apple Seedlings

The previous results showed that strain LW-66 displayed strong preventive effects against *V. mali* on detached apple branches. However, it is not known if *Bacillus* sp. LW-66 also has control efficacy against *V. mali* on apple trees already infected with the plant pathogen. Therefore, this study further analyzed the control efficacy of *Bacillus* sp. LW-66 against *V. mali* utilizing a potted apple seedling infection model. The results are shown in Table 7. After 30 days of treatment, *V. mali*-infected potted apple seedlings treated with the bone-glue bacterial agent of *Bacillus* sp. LW-66 (adjusted to 1 × 10^9^ CFU/mL) demonstrated a survival rate as high as 90%. In contrast, the survival rates of seedlings treated with the matrix control and the untreated control were only 35% and 30%, respectively. These results indicate that the bone-glue bacterial agent prepared from *Bacillus* sp. LW-66 has substantial control efficacy against *V. mali*.

### 3.11. Bioinformatics Analysis of Bacillus *sp*. LW-66

#### 3.11.1. Genome Sequencing and Species Annotation

**Figure 8:**
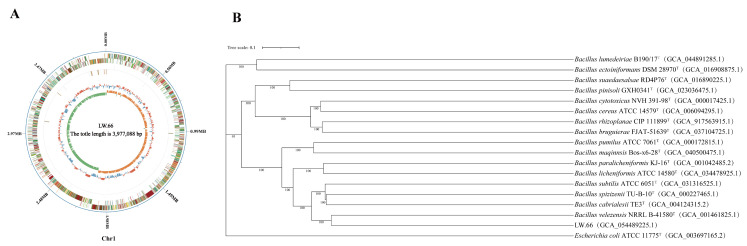
Circular genome map of *Bacillus* sp. LW-66. (**A**): Circular genome map of *Bacillus* sp. LW-66. Circles (outer to inner): genome coordinates, COG/KOG genes, ncRNA, GC content, GC skew. (**B**): Phylogenetic tree of *Bacillus* sp. LW-66 based on the whole-genome sequence.

We sequenced and assembled the genome of *Bacillus* sp. LW-66, obtaining a single linear chromosome of 3,977,088 bp (Figure 8A). The contig had a G+C content of 46.45% and an N50 of 16,956 bp. Gene prediction demonstrated 4063 coding sequences, totaling 3,584,733 bp (average 882 bp), covering 90.13% of the genome. We also found 86 tRNA genes, 27 rRNA genes, 12 sRNA genes, 88 minisatellites, 1 microsatellite, 142 repetitive sequences, and 104 tandem repeats (lengths 6–282 bp, sum 6369 bp). The latter accounted for 0.1601% of the genome. The raw genome sequence results of *Bacillus* sp. LW-66 have been deposited in NCBI under the accession number: GCA_054489225.1.

The NR database is a non-redundant protein database that can be employed for taxonomic classification. In this study, the amino acid sequences of all predicted proteins from *Bacillus* sp. LW-66 were aligned against the NR database using Diamond software to conduct gene annotation and species assignment. Statistical analysis of the alignment results revealed that *Bacillus* sp. LW-66 exhibited the highest degree of sequence similarity to *B. velezensis*, with 1701 protein-coding genes in its genome being annotated to *B. velezensis*.

To further clarify its taxonomic status, we ran genome-wide comparisons and similarity analyses on TYGS, and a maximum-likelihood phylogenetic tree was generated (Figure 8B). Using the Genome BLAST Distance Phylogeny (GBDP) pipeline implemented in the DSMZ GGDC online server (version 3.0), digital DNA-DNA hybridization (dDDH) scores were computed between *Bacillus* sp. LW-66 and its closely related phylogenetic neighbors (Table S4). The analysis demonstrated that *Bacillus* sp. LW-66 shared the closest genomic relationship with *B. velezensis* NRRL B-41580ᵀ (assembly accession: GCA_001461825.1), with a dDDH value of 94.9%. As dDDH values > 70% are widely accepted as the threshold for species delineation in prokaryotes, this high value confirms that *Bacillus* sp. LW-66 belongs to the species *B. velezensis*. Therefore, we formally reclassified and named this strain as *B. velezensis* LW-66.

#### 3.11.2. Prediction and Analysis of Secondary Metabolite Biosynthetic Gene Clusters

We used antiSMASH 8.0.4 to analyze the genome of *B. velezensis* LW-66. In total, 13 distinct secondary metabolite biosynthetic gene clusters were identified, covering a diverse array of metabolite classes, including non-ribosomal peptide synthetases (NRPS), polyketide synthases (PKS), and terpenes (Table 8). Among these, seven gene clusters displayed high homology (similarity ≥ 92%) to known biosynthetic gene clusters responsible for biocontrol-related antifungal substances. The fengycin biosynthetic gene cluster produces fengycin, a signature antifungal metabolite of *B. velezensis* that specifically disrupts the phospholipid bilayer of fungal cell membranes, resulting in swelling, thickening, rupture, and death of fungal mycelial cells. This mechanism is consistent with the observed morphological abnormalities (e.g., significant thickening and fragmentation) in *V. mali* hyphae following antagonism by *B. velezensis* LW-66 in this study, implying that the LW.66_GM002164–LW.66_GM002235 gene cluster may be responsible for these hyphal alterations.

## 4. Discussion

In this study, *B. velezensis* strain LW-66 was isolated from a soil sample and exhibited potent antagonistic activity against the phytopathogen *V. mali*. Combining morphological characterization, 16S rRNA gene sequencing, and whole-genome analysis, the isolate was taxonomically identified as *B. velezensis* and designated as LW-66.

Employing antagonistic microorganisms to control plant diseases is an effective strategy for achieving green and sustainable agricultural development. Among these, *B. velezensis* has attracted substantial attention due to the several advantages it offers, including growth promotion and broad-spectrum antifungal activity [50,51]. This strain exerts its biocontrol activity through multiple synergistic mechanisms, including the production of antibiotics and other antimicrobial metabolites (e.g., lipopeptides and polyketides) that inhibit the growth and metabolic processes of pathogenic fungi [52,53]; the synthesis and secretion of plant hormones to compete for nutrients, such as iron, to enhance plant resistance to diseases and block pathogenic infections [54,55,56,57]; and induction of systemic resistance in plants, thereby strengthening plant defense responses [58,59]. However, the biocontrol efficacy of different *B. velezensis* strains varies significantly. Screening strains that have both high antifungal activity and strong environmental tolerance is crucial for their development for field application. The LW-66 strain isolated in this study not only possesses the typical multi-mechanism antifungal characteristics of *B. velezensis* but also demonstrates promising potential for field application. Our experiments showed that LW-66 has significant inhibitory effects against five plant pathogenic fungi. Active extracts of LW-66 maintained stable antifungal activity within the temperature range of 25–90 °C and pH range of 2–8, with only a slight decrease in activity under extreme conditions of 100 °C or pH ≥ 10.

To further verify its biocontrol potential in practical scenarios, detached apple branches and potted seedling assays were performed in this study. Both the bacterial strain *Bacillus* sp. LW-66 and active extract achieved control efficacies exceeding 90% in detached branches. In potted experiments, LW-66 was prepared into a biocontrol agent using an existing bone-glue agent as the carrier, and the survival rate of apple seedlings reached 90%. Bone glue exhibits strong adhesion, reduces bacterial desiccation, and has good biocompatibility, which can improve the colonization and long-lasting efficacy of LW-66 on plant surfaces [60,61,62]. Additionally, LW-66 produces proteases, amylases, catalase, and oxidase, which may enhance antifungal activity by damaging pathogen mycelia [63]. Strain LW-66 also has advantages of easy culture, short fermentation period, stable metabolites, and low production cost. Collectively, these findings demonstrate that strain LW-66 exhibits robust environmental adaptability and outstanding biocontrol potential.

To elucidate the molecular basis underlying its antifungal activity, genomic analysis was performed to identify the antifungal substances synthesized by LW-66. Analysis utilizing the antiSMASH system revealed that the genome of LW-66 contains 13 secondary metabolite biosynthetic gene clusters. Of these gene clusters, seven exhibited high homology (similarity ≥ 92%) to known biosynthetic gene clusters responsible for biocontrol-related antifungal compounds. The corresponding products were surfactin, fengycin, bacillaene, macrolactin H, difficidin, bacilysin, and bacillibactin [64]. Among these, fengycin, an antifungal lipopeptide, has been shown to specifically disrupt fungal cell membranes, leading to hyphal swelling and rupture [52,65,66]. This aligns directly with the morphological abnormalities (thickening and fragmentation) observed in *V. mali* hyphae following antagonism by LW-66 in this study, indicating that this compound is likely a key contributor to the antifungal activity of LW-66. Surfactin possesses the dual functions of direct antifungal activity and enhanced colonization. The amphiphilic structure of surfactin is able not only to disrupt pathogen cell membranes but also to decrease the liquid surface tension, which, in turn, improves the adhesion ability of LW-66 on apple branch surfaces. This likely explains the sustained control efficacy observed in the potted seedling experiments [52,67,68]. Difficidin and macrolactin H exert antifungal activity by disrupting the ergosterol biosynthesis pathway in fungi. Additionally, bacillibactin competitively inhibits pathogen growth by chelating environmental iron ions [63].

Notably, LW-66 produces volatile organic compounds (VOCs) with broad antifungal effects, and its VOCs inhibited *V. mali* by 93.27%, indicating that this strain has non-contact antifungal activity that may suppress disease spread via air diffusion under field conditions. Compared with commercial strains FZB42, QST713, and 83, LW-66 possesses three terpene biosynthetic gene clusters, which are more abundant than those in other strains. Terpenoid compounds, especially monoterpenes and their derivatives, can exert antifungal effects through gas-phase diffusion without direct contact with pathogens, which may serve as the key molecular basis for the volatile antifungal activity of LW-66 [50,69,70,71]. Furthermore, LW-66 contains two bacillaene gene clusters, whereas these three commercial strains each harbor only one. As a polyketide antibiotic, bacillaene functions by inhibiting ribosomal elongation factors in pathogens, and the presence of duplicate gene clusters may improve its synthetic efficiency or yield [63,72,73,74]. These features collectively make LW-66 distinctive and may contribute to its strong antifungal activity.

Despite these promising results, this study has several limitations that should be addressed in future research: (1) biocontrol efficacy was only evaluated using detached branches and potted seedlings, without field validation; (2) the long-term colonization and survival of LW-66 in complex field environments remain unclear; (3) ecological safety was not assessed, including impacts on non-target organisms and soil microbial communities; (4) active antifungal compounds were predicted genomically but not structurally identified by LC-MS; (5) the chemical compositions of volatile antifungal metabolites were not characterized, and (6) the bone-glue bacterial agent was prepared based on previous reports without independent optimization of its components and proportion.

In conclusion, *B. velezensis* LW-66 is a promising biocontrol strain with broad-spectrum antifungal activity, favorable environmental stability, and strong volatile-mediated inhibition. It is suitable for development as a biological agent to control fungal diseases such as apple *Valsa* canker. Future studies should focus on identifying key antifungal compounds and their regulatory mechanisms, conducting systematic field trials, evaluating ecological safety, and performing systematic optimization of the bone-glue bacterial agent to improve its stability and practical application effect, so as to support the practical application of this strain.

## 5. Conclusions

*B. velezensis* LW-66 exhibits strong broad-spectrum antifungal activity and favorable environmental stability, demonstrating excellent biocontrol efficacy against *V. mali* in both detached branch and potted seedling assays. Genomic analysis identified three terpene clusters and duplicated bacillaene clusters as unique features distinguishing it from commercial strains, which likely contributed to its potent antagonism and volatile-mediated non-contact inhibition. Future studies will focus on field trials, chemical identification of key antifungal compounds and active VOCs, formulation optimization, and ecological safety assessment to further promote its practical application.

## Figures and Tables

**Table 1:** Physiological and biochemical characteristics of strain LW-66.

Type	Result	Type	Result
V-P	− (no red color development after reagent addition; medium remained yellow)	3% NaCL	+ (visible bacterial growth)
Methyl red	+ (medium turned bright red after indicator addition)	5% NaCL	+ (visible bacterial growth)
Oxidase	+ (filter paper turned dark blue/purple within 10 s)	7% NaCL	+ (visible bacterial growth)
Catalase	+ (immediate and vigorous bubble formation after H_2_O_2_ addition)	10% NaCL	+ (visible bacterial growth)
Glucose fermentation	+ (medium turned yellow, indicating acid production)	15% NaCL	− (no visible bacterial growth)
Lactose fermentation	− (medium remained purple, no acid production)	Maltose fermentation	− (medium remained purple, indicating no acid production)
Fructose fermentation	+ (medium turned yellow, indicating acid production)	Sucrose fermentation	+ (medium turned yellow, indicating acid production)

Note: “+“ means positive and “−“ means negative.

**Table 2:** Inhibitory effect of different concentrations of *Bacillus* sp. LW-66 cell-free filtrate on the mycelial growth of *V. mali*.

Filtrate Concentration/%	Colony Diameter/mm	Disease Prevention Effect/%
0%	82.00 ± 0.87 d	0.00 ± 0.00 a
1%	81.17 ± 0.58 d	1.13 ± 0.78 a
2%	77.83 ± 0.76 c	5.63 ± 1.03 b
4%	72.67 ± 1.04 b	12.61 ± 1.41 c
8%	63.33 ± 0.76 a	25.23 ± 1.03 d
16%	62.00 ± 0.87 a	27.03 ± 1.17 e

Note: Values in the same column with different lowercase letters differ significantly (*p* < 0.05). One-way ANOVA results: for colony diameter, *F* = 339.820, df = 5, 12, *p* < 0.001; for Disease prevention effect, *F* = 416.280, df = 5, 12, *p* < 0.001.

**Table 3:** Stability of the active extract of *Bacillus* sp. LW-66 at various temperature levels.

T (°C)	Colony Diameter/mm	Disease Prevention Effect/%
25	19.50 ± 1.32 a	84.56 ± 1.78 a
50	19.33 ± 0.76 a	84.79 ± 1.03 a
60	20.83 ± 1.04 ab	82.77 ± 1.40 ab
70	22.33 ± 1.04 b	80.76 ± 1.40 b
80	24.17 ± 0.76 c	78.30 ± 1.03 c
90	27.50 ± 1.00 d	73.83 ± 1.34 d
100	35.83 ± 1.04 e	62.64 ± 1.40 e
CK	82.50 ± 1.00 f	-

Note: Values in the same column with different lowercase letters differ significantly (*p* < 0.05). One-way ANOVA results: for colony diameter, *F* = 1334.787, df = 7,16, *p* < 0.001; for Disease prevention effect, *F* = 101.004, df = 6, 14, *p* < 0.001.

**Table 4:** Stability of the active extract of *Bacillus* sp. LW-66 at various pH levels.

pH	Colony Diameter/mm	Disease Prevention Effect/%
2	22.17 ± 0.58 b	80.86 ± 0.78 bc
4	20.83 ± 0.29 ab	82.66 ± 0.39 ab
6	19.83 ± 0.76 a	84.01 ± 1.03 a
8	23.83 ± 0.29 c	78.60 ± 0.39 c
10	32.83 ± 0.58 d	66.44 ± 0.78 d
12	39.67 ± 2.25 e	57.21 ± 3.05 e
CK1	82.00 ± 0.87 f	-
CK2	81.83 ± 0.29 f	-

Note: Values in the same column with different lowercase letters differ significantly (*p* < 0.05). CK1: 1 mol/L HCl; CK2: 1 mol/L NaOH. One-way ANOVA results: for colony diameter, *F* = 2299.240, df = 7,16, *p* < 0.001; for Disease prevention effect, *F* = 174,967, df = 5, 12, *p* < 0.001.

**Table 5:** Effect of *Bacillus* sp. LW-66 on the prevention of *V. mali* infection in apple tree branches.

Type	Average Length of Lesions (mm)	Average Width of Lesions (mm)	Lesion Area (mm^2^)	Disease Prevention Effect (%)
LW-66	9.33 ± 1.15 a	7.33 ± 1.53 a	53.93 ± 14.60 a	91.29 ± 2.36
CK	33.33 ± 1.53 e	23.67 ± 1.15 d	618.89 ± 19.82 d	

Note: Values in the same column with different lowercase letters differ significantly (*p* < 0.05). One-way ANOVA results: for lesion length, *F* = 471.273, df = 1, 4, *p* < 0.001; for lesion width, *F* = 218.273, df = 1, 4, *p* < 0.001; for lesion area, *F* = 1579.922, df = 1, 4, *p* < 0.001.

**Table 6:** Preventive Effect of Active Extracts from *Bacillus* sp. LW-66 against *V*. *mali* Infection in Apple Tree Branches.

Type	Diluted Times	Average Length of Lesions (mm)	Average Width of Lesions (mm)	Lesion Area (mm^2^)	Disease Prevention Effect (%)
Active extract	1	9.00 ± 1.00 a	7.00 ± 1.00 a	50.00 ± 12.57 a	92.21 ± 1.96 c
	1/2	10.67 ± 1.15 a	6.67 ± 1.53 a	56.03 ± 14.60 a	91.27 ± 2.27 c
	1/5	15.67 ± 2.52 b	7.00 ± 1.00 a	86.66 ± 22.98 ab	86.50 ± 3.58 bc
	1/10	17.33 ± 1.15 bc	7.33 ± 1.53 a	100.53 ± 25.95 ab	84.34 ± 4.04 bc
	1/20	19.00 ± 1.00 c	8.33 ± 1.15 ab	124.35 ± 18.62 b	80.63 ± 2.90 b
	1/50	28.00 ± 3.00 d	10.33 ± 1.53 bc	226.46 ± 34.36 c	64.72 ± 5.35 a
	1/100	29.67 ± 0.58 d	12.00 ± 2.65 c	280.13 ± 65.40 c	56.36 ± 10.19 a
CK	-	35.00 ± 1.00 e	23.33 ± 1.53 d	641.93 ± 56.42 d	

Note: Values in the same column with different lowercase letters differ significantly (*p* < 0.05) One-way ANOVA results: for lesion length, *F* = 98.855, df = 7, 16, *p* < 0.001; for lesion width, *F* = 38.421, df = 7, 16, *p* < 0.001; for lesion area, *F* = 88.946, df = 7, 16, *p* < 0.001; for control efficacy, *F* = 22.043, df = 6, 14, *p* < 0.001.

**Table 7:** Control effect of the bone-glue bacterial agent on apple Valsa canker after 30 days of treatment.

Processing Method	Infected Trees	Dead Trees	Surviving Trees	Survival Rate (%)
Control	20	14	6	30
Basic material	20	13	7	35
Bone-glue bacterial agent	20	2	18	90

**Table 8:** Secondary metabolite gene clusters of *B. velezensis* LW-66.

Clusters	Type	Gene ID	Similar Known Cluster	Gene Number	Similarity (%)	
Cluster 1	Other	LW.66_GM000324-LW.66_GM000370	Bacilysin	46	98	
Cluster 2	Ripp-like, NRPS	LW.66_GM000944- LW.66_GM001010	Bacillibactin	66	100	
Cluster 3	Terpene-precursor	LW.66_GM001620- LW.66_GM001645	–	25	–	
Cluster 4	transAT-PKS	LW.66_GM001672- LW.66_GM001733	Difficidin	61	95	
Cluster 5	T3PKS	LW.66_GM001973- LW.66_GM001984	–	11	–	
Cluster 6	terpene	LW.66_GM002122- LW.66_GM002133	–	11	–	
Cluster 7	NRPS, betalactone, transAT-PKS	LW.66_GM002164- LW.66_GM002235	Fengycin	71	100	
Cluster 8	transAT-PKS, NRPS, T3PKS	LW.66_GM002310- LW.66_GM002365	Bacillaene	55	97	
Cluster 9	transAT-PKS	LW.66_GM002589- LW.66_GM002636	Macrolactin H	47	92	
Cluster 10	Terpene	LW.66_GM002973- LW.66_GM002996	–	23	–	
Cluster 11	PKS-like	LW.66_GM003082- LW.66_GM003125	–	43	–	
Cluster 12	T3PKS, transAT-PKS, T1PKS	LW.66_GM003341- LW.66_GM003392	Bacillaene	51	65	
Cluster 13	NRPS	LW.66_GM003700- LW.66_GM003744	Surfactin	44	100	

## Data Availability

The original contributions presented in this study are included in the article and Supplementary Materials. Further inquiries can be directed to the corresponding authors.

## Supplementary Materials

The following supporting information can be downloaded at: https://www.mdpi.com/article/10.3390/microorganisms14040889/s1, Table S1: The inhibitory rates of the strain LW-66 against *V. mali*.; Table S2: Inhibitory effect of the strain LW-66 on the growth of five plant pathogenic fungi on PDA plates; Table S3: Inhibitory effects of volatile substances from strain LW-66 on the mycelial growth of five plant pathogenic fungi; Table S4: Genomic characteristics of *Bacillus* sp. LW-66 phylogenetic tree members [75,76,77,78,79,80,81,82,83,84,85,86,87,88], Figure S1. Analysis of the extracellular enzyme activities of strain LW-66.

## References

[1] Liu H., Wang H., Liao X.L., Gao B., Lu X., Sun D., Gong W., Zhong J., Zhu H., Pan X. (2022). Mycoviral gene integration converts a plant pathogenic fungus into a biocontrol agent. Proc. Natl. Acad. Sci. USA.

[2] Le K.D., Yu N.H., Park A.R., Park D.J., Kim C.J., Kim J.C. (2022). *Streptomyces* sp. AN090126 as a Biocontrol Agent against Bacterial and Fungal Plant Diseases. Microorganisms.

[3] Meng X.L., Yang R., Liu A.T., Hu T.L., Wang Y.N., Cao K.Q., Wang S.T. (2021). The Influence of Lower Temperature Induction of *Valsa mali* on the Infection of Apple Trees. Plant Dis..

[4] Kiani T., Mehboob F., Hyder M.Z., Zainy Z., Xu L., Huang L., Farrakh S. (2021). Control of stripe rust of wheat using indigenous endophytic bacteria at seedling and adult plant stage. Sci. Rep..

[5] Sosa-Zuniga V., Vidal Valenzuela Á., Barba P., Espinoza Cancino C., Romero-Romero J.L., Arce-Johnson P. (2022). Powdery Mildew Resistance Genes in Vines: An Opportunity to Achieve a More Sustainable Viticulture. Pathogens.

[6] Munir M., Smith H., Valentine T., Leonberger K., Szarka D., Dixon E., Anthony N., Ricciardi M., Adedokun T., Keene T. (2024). Leaf Spot Disease Development and Its Effect on Yield of Essential Oil-Producing Hemp Cultivars in Kentucky. Plant Dis..

[7] Sun Q., Fei S., Huang S., Tan R., Chen H., Song S., Wang B. (2025). Wilt disease reshapes rhizosphere microbiota in small yellow ginger soils. Front. Microbiol..

[8] Zhang H., Cheng J.L., Zhu X.F., Zhang S.L., Yan L.W., Lin J.S. (2022). Identification and biocontrol evaluation of *Streptomyces* sp. strain ZH-356 antagonistic to plant pathogenic fungi. Acta Microbiol. Sin..

[9] Gutter Y., Shachnai A., Schiffmann-Nadel M., Dinoor A. (1981). Chemical control in citrus of green and blue molds resistant to benzimidazoles. J. Phytopathol..

[10] Moonjely S., Ebert M., Paton-Glassbrook D., Noel Z.A., Roze L., Shay R., Watkins T., Trail F. (2023). Update on the state of research to manage Fusarium head blight. Fungal Genet. Biol..

[11] Bardas G.A., Veloukas T., Koutita O., Karaoglanidis G.S. (2010). Multiple resistance of Botrytis cinerea from kiwifruit to SDHIs, QoIs and fungicides of other chemical groups. Pest Manag. Sci..

[12] Bai Y.B., Gao Y.Q., Nie X.D., Tuong T.M., Li D., Gao J.M. (2019). Antifungal Activity of Griseofulvin Derivatives against Phytopathogenic Fungi in Vitro and in Vivo and Three-Dimensional Quantitative Structure-Activity Relationship Analysis. J. Agric. Food Chem..

[13] Song B., Zhang T.F., Yang C.X., Wen G.Q. (2022). Preliminary study on biocontrol of *Bacillus velezensis* against *Penicillium crustosum* in citrus. Southwest China J. Agric. Sci..

[14] Zhu X.F., Ning W.Q., Xiao W., Wang Z.R., Li S.L., Zhang J.L., Ren M., Xu C.N., Liu B., Wang Y.F. (2025). Isolation and Identification of Burkholderia stagnalis YJ-2 from the Rhizosphere Soil of Woodsia ilvensis to Explore Its Potential as a Biocontrol Agent Against Plant Fungal Diseases. Microorganisms.

[15] Dhouib H., Zouari I., Ben Abdallah D., Belbahri L., Taktak W., Triki M.A., Tounsi S. (2019). Potential of a novel endophytic *Bacillus velezensis* in tomato growth promotion and protection against Verticillium wilt disease. Biol. Control.

[16] Kanjanamaneesathian M., Wiwattanapatapee R., Rotniam W., Pengnoo A., Wongpetkhiew W., Tanmala V. (2013). Application of a suspension concentrate formulation of *Bacillus velezensis* to control root rot of hydroponicallygrown vegetables. New Zealand Plant Prot..

[17] Fira D., Dimkić I., Berić T., Lozo J., Stanković S. (2018). Biological control of plant pathogens by *Bacillus* species. J. Biotechnol..

[18] Yang F., Zhang R., Wu X., Xu T., Ahmad S., Zhang X., Zhao J., Liu Y. (2020). An endophytic strain of the genus *Bacillus* isolated from the seeds of maize (*Zea mays* L.) has antagonistic activity against maize pathogenic strains. Microb. Pathog..

[19] Azabou M.C., Gharbi Y., Medhioub I., Ennouri K., Barham H., Tounsi S., Triki M.A. (2020). The endophytic strain *Bacillus velezensis* OEE1: An efficient biocontrol agent against Verticillium wilt of olive and a potential plant growth promoting bacteria. Biol. Control.

[20] Xu W., Zhang L., Goodwin P.H., Xia M., Zhang J., Wang Q., Liang J., Sun R., Wu C., Yang L. (2020). Isolation, Identification, and Complete Genome Assembly of an Endophytic *Bacillus velezensis* YB-130, Potential Biocontrol Agent Against Fusarium graminearum. Front. Microbiol..

[21] Yan Y., Xu W., Hu Y., Tian R., Wang Z. (2022). *Bacillus velezensis* YYC promotes tomato growth and induces resistance against bacterial wilt. Biol. Control.

[22] Wang J.Z., Mao Z.Q., Cong P.H., Lü D.G., Ma F.W., Ren X.L., Shu H.R., Li B.H., Guo Y.R., Hao Y.J. (2019). Fruit scientific research in New China in the past 70 years: Apple. J. Fruit Sci..

[23] Xu W., Sun H.Y., Jin J.W., Cheng J.M. (2020). Predicting the potential distribution of apple canker pathogen (*Valsa mali*) in China under climate change. Forests.

[24] Wang L., Zang R., Huang L.L., Xie F.Q., Gao X.N. (2005). The investigation of apple tree Valsa canker in Guanzhong region of Shaanxi province. J. Northwest Sci-Tech Univ. Agric. For..

[25] Peng H.X., Wei X.Y., Xiao Y.X., Sun Y., Biggs A.R., Gleason M.L., Shang S.P., Zhu M.Q., Guo Y.Z., Sun G.Y. (2016). Management of Valsa Canker on Apple with Adjustments to Potassium Nutrition. Plant Dis..

[26] Chen C., Li B.H., Dong X.L., Wang C.X., Lian S., Liang W.X. (2016). Effects of Temperature, Humidity, and Wound Age on *Valsa mali* Infection of Apple Shoot Pruning Wounds. Plant Dis..

[27] Du Y., Jia H., Yang Z., Wang S., Liu Y., Ma H., Liang X., Wang B., Zhu M., Meng Y. (2023). Sufficient coumarin accumulation improves apple resistance toCytospora maliunder high-potassium status. Plant Physiol..

[28] Feng H., Wang C., He Y., Tang L., Han P., Liang J., Huang L. (2023). Apple Valsa canker: Insights into pathogenesis and disease control. Phytopathol. Res..

[29] Zhang S., Chen X., Duan C., Liu J., Tao F., Xu B. (2025). Identification, Subcellular Localization, and Infection-Related Expression of a Novel Haloacid Dehalogenase Gene (*VmHAD*) from *Valsa mali* Vm1. J. Fungi.

[30] Zhang J.X., Gu Y.B., Chi F.M., Ji Z.R., Wu J.Y., Dong Q.L., Zhou Z.S. (2015). *Bacillus amyloliquefaciens* GB1 can effectively control apple valsa canker. Biol. Control.

[31] Sun L., Gao J., Tan Y., Xia Y., Wang R., Liu R., Zhang X., Yan X., Huang L. (2026). Biocontrol of Apple Valsa Canker by *Bacillus* sp. H12 and modulation of the apple seedlings microbiome. Pestic. Biochem. Physiol..

[32] Xue Y.Y., Li X., Li F.K., Gou P.N., Li L., Xu B.L. (2021). Identification of Z-12A fungus for biocontrol of apple tree canker. Microbiol. China.

[33] Xu L., Meng Y., Liu R., Xiao Y., Wang Y., Huang L. (2023). Inhibitory effects of *Bacillus vallismortis* T27 against apple Valsa canker caused by *Valsa mali*. Pestic. Biochem. Physiol..

[34] Wang H., Guo J., Chen X., He H. (2023). The Metabolomics Changes in Luria–Bertani Broth Medium under Different Sterilization Methods and Their Effects on *Bacillus* Growth. Metabolites.

[35] Wang X., Li Q., Sui J., Zhang J., Liu Z., Du J., Xu R., Zhou Y., Liu X. (2019). Isolation and Characterization of Antagonistic Bacteria *Paenibacillus jamilae* HS-26 and Their Effects on Plant Growth. BioMed Res. Int..

[36] Han H.J., Ko M.N., Shin C.S., Hyun C.G. (2024). Human health benefits and microbial consortium of stevia fermented with barley nuruk. Fermentation.

[37] O’Reilly T., Niven D.F. (1986). Tryptone-yeast extract broth as a culture medium for Haemophilus pleuropneumoniae and Haemophilus parasuis to be used as challenge inocula. Can. J. Vet. Res..

[38] Dong X.Z., Cai M.Y. (2001). Manual for the Identification of Common Bacterial Systems.

[39] Logan N.A., De Vos P., Whitman W.B. (2015). Bacillus. Bergey’s Manual of Systematics of Archaea and Bacteria.

[40] Zou Y., Zhang Z., Zeng Y., Hu H., Hao Y., Huang S., Li B. (2024). Common Methods for Phylogenetic Tree Construction and Their Implementation in R. Bioengineering.

[41] Zhang Y.-C., Zhan X., Chen J.-Y., Yu D.-T., Zhang T., Zhang H., Duan C.-G. (2025). Reduced fungal protein acetylation mediates the antimicrobial activity of a rhizosphere bacterium against a phytopathogenic fungus. Nat. Commun..

[42] Zhou D., Jing T., Chen Y., Yun T., Qi D., Zang X., Zhang M., Wei Y., Li K., Zhao Y. (2022). Biocontrol potential of a newly isolated *Streptomyces* sp. HSL-9B from mangrove forest on postharvest anthracnose of mango fruit caused by Colletotrichum gloeosporioides. Food Control.

[43] Ghyselinck J., Coorevits A., Van Landschoot A., Samyn E., Heylen K., De Vos P. (2013). An rpoD gene sequence based evaluation of cultured Pseudomonas diversity on different growth media. Microbiology.

[44] Meng Y., Li J., Yuan W., Liu R., Xu L., Huang L. (2024). *Pseudomonas thivervalensis* K321, a promising and effective biocontrol agent for managing apple Valsa canker triggered by *Valsa mali*. Pestic. Biochem. Physiol..

[45] Xu L.B., Di C.X., Liu A.P., Wulanbateer, Zhao H.X. (2010). Antibacterial Characteristics of Actinomycete Strain NMG8-2. Acta Agric. Boreali-Sin..

[46] Lai W., Liang Y., Li X., Long C.-a. (2026). Biocontrol efficacy and mechanisms of *Bacillus proteolyticus* L181 against postharvest sour rot of citrus. Postharvest Biol. Technol..

[47] Li X., Yuan H., Shi B., Liu R., Chen Y., Wang L., Tu H., Hou H. (2026). Antifungal mechanism of Aspergillus tubingensis strain Pa6 against strawberry gray mold and its application in the preservation of postharvest strawberry. Postharvest Biol. Technol..

[48] Mao W.X., Li Y., Zhang S.W., Xue Y.Y., Xu B.L. (2019). Evaluation of indoor control efficacy of five botanical agents against *Valsa mali*. Plant Prot..

[49] Liu A.D., Ning W.Q., Zhu X.F., Zhang B.L., Qu X.N., Zhang J.L., Xu C.N., Li G.W., Zhang X.Q., Wang Y.F. (2025). Biocontrol effects of *Streptomyces* sp. ZH-356 on plant diseases. Acta Microbiol. Sin..

[50] Lim S.M., Yoon M.Y., Choi G.J., Choi Y.H., Jang K.S., Shin T.S., Park H.W., Yu N.H., Kim Y.H., Kim J.C. (2017). Diffusible and Volatile Antifungal Compounds Produced by an Antagonistic *Bacillus velezensis* G341 against Various Phytopathogenic Fungi. Plant Pathol. J..

[51] Jin Q., Jiang Q., Zhao L., Su C., Li S., Si F., Li S., Zhou C., Mu Y., Xiao M. (2017). Complete genome sequence of *Bacillus velezensis* S3-1, a potential biological pesticide with plant pathogen inhibiting and plant promoting capabilities. J. Biotechnol..

[52] Grady E.N., MacDonald J., Ho M.T., Weselowski B., McDowell T., Solomon O., Renaud J., Yuan Z.C. (2019). Characterization and complete genome analysis of the surfactin-producing, plant-protecting bacterium *Bacillus velezensis* 9D-6. BMC Microbiol..

[53] Rabbee M.F., Ali M.S., Choi J., Hwang B.S., Jeong S.C., Baek K.H. (2019). *Bacillus velezensis*: A Valuable Member of Bioactive Molecules within Plant Microbiomes. Molecules.

[54] Zhu L., Huang J., Lu X., Zhou C. (2022). Development of plant systemic resistance by beneficial rhizobacteria: Recognition, initiation, elicitation and regulation. Front. Plant Sci..

[55] Choudhary D.K., Johri B.N. (2009). Interactions of *Bacillus* spp. and plants--with special reference to induced systemic resistance (ISR). Microbiol. Res..

[56] Kang X., Zhang W., Cai X., Zhu T., Xue Y., Liu C. (2018). *Bacillus velezensis* CC09: A Potential ‘Vaccine’ for Controlling Wheat Diseases. Mol. Plant-Microbe Interact. MPMI.

[57] Xu Z., Mandic-Mulec I., Zhang H., Liu Y., Sun X., Feng H., Xun W., Zhang N., Shen Q., Zhang R. (2019). Antibiotic Bacillomycin D Affects Iron Acquisition and Biofilm Formation in *Bacillus velezensis* through a Btr-Mediated FeuABC-Dependent Pathway. Cell Rep..

[58] Rabbee M.F., Hwang B.S., Baek K.H. (2023). *Bacillus velezensis*: A beneficial biocontrol agent or facultative phytopathogen for sustainable agriculture. Agronomy.

[59] Pan L., Zhao X., Chen M., Fu Y., Xiang M., Chen J. (2020). Effect of exogenous methyl jasmonate treatment on disease resistance of postharvest kiwifruit. Food Chem..

[60] Park S., Safdar M., Kim W., Seol J., Kim D., Lee K.H., Son H.I., Kim J. (2024). Gelatin Nanoparticles can Improve Pesticide Delivery Performance to Plants. Small.

[61] Kobetičová K., Böhm M., Jerman M., Dušek J., Černý R. (2022). Ecotoxicity and Biodegradation of Sustainable Environment-Friendly Bone-Glue-Based Adhesive Suitable for Insulation Materials. Polymers.

[62] Silva G.M.d., Santos N.C., Silva L.A.d., de Lima T.L.B., Leite M.d.O., Silva V.M.d.A., Oliveira L.d.S., Ribeiro V.H.d.A., Meira A.S., Felix P.H.D. (2025). Fish Gelatin Edible Films with Prebiotics and Structuring Polysaccharides for Probiotic Delivery: Physicochemical Properties, Viability, and In Vitro Gastrointestinal Release. Polysaccharides.

[63] Fazle Rabbee M., Baek K.H. (2020). Antimicrobial Activities of Lipopeptides and Polyketides of *Bacillus velezensis* for Agricultural Applications. Molecules.

[64] Meena K.R., Kanwar S.S. (2015). Lipopeptides as the antifungal and antibacterial agents: Applications in food safety and therapeutics. Biomed Res. Int..

[65] Wood T.M., Martin N.I. (2019). The calcium-dependent lipopeptide antibiotics: Structure, mechanism, & medicinal chemistry. MedChemComm.

[66] Li B., Li Q., Xu Z., Zhang N., Shen Q., Zhang R. (2014). Responses of beneficial *Bacillus amyloliquefaciens* SQR9 to different soilborne fungal pathogens through the alteration of antifungal compounds production. Front. Microbiol..

[67] Zhou D., Hu F., Lin J., Wang W., Li S. (2019). Genome and transcriptome analysis of *Bacillus velezensis* BS-37, an efficient surfactin producer from glycerol, in response to d-/L-leucine. MicrobiologyOpen.

[68] Bais H.P., Fall R., Vivanco J.M. (2004). Biocontrol of *Bacillus subtilis* against infection of Arabidopsis roots by Pseudomonas syringae is facilitated by biofilm formation and surfactin production. Plant Physiol..

[69] De la Cruz-López N., Cruz-López L., Holguín-Meléndez F., Guillén-Navarro G.K., Huerta-Palacios G. (2022). Volatile Organic Compounds Produced by Cacao Endophytic Bacteria and Their Inhibitory Activity on Moniliophthora roreri. Curr. Microbiol..

[70] Stamenković J.G., Petrović G.M., Đorđević A.S. (2022). Phytochemical Analysis and Antibacterial Activity of *Achillea coarctata* Poir. Essential Oils. Chem. Biodivers..

[71] Giorgio A., De Stradis A., Lo Cantore P., Iacobellis N.S. (2015). Biocide effects of volatile organic compounds produced by potential biocontrol rhizobacteria on Sclerotinia sclerotiorum. Front. Microbiol..

[72] Pandin C., Le Coq D., Deschamps J., Védie R., Rousseau T., Aymerich S., Briandet R. (2018). Complete genome sequence of *Bacillus velezensis* QST713: A biocontrol agent that protects *Agaricus bisporus* crops against the green mould disease. J. Biotechnol..

[73] Balderas-Ruíz K.A., Bustos P., Santamaria R.I., González V., Cristiano-Fajardo S.A., Barrera-Ortíz S., Mezo-Villalobos M., Aranda-Ocampo S., Guevara-García Á.A., Galindo E. (2020). *Bacillus velezensis* 83 a bacterial strain from mango phyllosphere, useful for biological control and plant growth promotion. AMB Express.

[74] Borriss R., Wu H., Gao X. (2019). Secondary metabolites of the plant growth promoting model rhizobacterium *Bacillus velezensis* FZB42 are involved in direct suppression of plant pathogens and in stimulation of plant-induced systemic resistance. Secondary Metabolites of Plant Growth Promoting Rhizomicroorganisms: Discovery and Applications.

[75] Liu G.H., Narsing Rao M.P., Shi H., Chen Q.Q., Quadri S.R., Li W.J. (2025). Description of six novel species *Bacillus bruguierae* sp. nov., *Bacillus kandeliae* sp. nov., *Bacillus yunxiaonensis* sp. nov., Metabacillus rhizosphaerae sp. nov., *Metabacillus sediminis* sp. nov. and *Psychrobacillus mangrovi* sp. nov., isolated from mangrove ecosystem. Int. J. Syst. Evol. Microbiol..

[76] Kämpfer P., Lipski A., McInroy J.A., Clermont D., Criscuolo A., Glaeser S.P. (2022). *Bacillus rhizoplanae* sp. nov. from maize roots. Int. J. Syst. Evol. Microbiol..

[77] Frankland G.C., Frankland P.F. (1887). XI. Studies on some new micro-organisms obtained from air. Philos. Trans. R. Soc. Lond. (B).

[78] Guinebretière M.H., Auger S., Galleron N., Contzen M., De Sarrau B., De Buyser M.L., Lamberet G., Fagerlund A., Granum P.E., Lereclus D. (2013). *Bacillus cytotoxicus* sp. nov. is a novel thermotolerant species of the *Bacillus cereus* Group occasionally associated with food poisoning. Int. J. Syst. Evol. Microbiol..

[79] Huang Y., Cai H., Qin S., Yang L., Zhou Y., Wu J., Chen X., Jiang M., Jiang Y., Ihsan Y.N. (2022). *Bacillus pinisoli* sp. nov., Isolated from Soil of a Decayed Pine Tree. Curr. Microbiol..

[80] Xu L., Huang X.X., Wang H.T., Tang S.K., Shen B., Sun J.Q. (2022). Description and characterization of three endophytic Bacillaceae from the halophyte Suaeda salsa: *Paenalkalicoccus suaedae* gen. nov., sp. nov., *Cytobacillus suaedae* sp. nov., and *Bacillus suaedae* sp. nov. Int. J. Syst. Evol. Microbiol..

[81] Zhu D., Zhang P., Niu L., Xie C., Li P., Sun J., Hang F. (2016). *Bacillus ectoiniformans* sp. nov., a halotolerant bacterium isolated from deep-sea sediments. Int. J. Syst. Evol. Microbiol..

[82] Costa L.V.D., Ramos J.N., Albuquerque L.S., Miranda R., Valadão T.B., Veras J.F.C., Vieira E.M.D., Forsythe S., Brandão M.L.L., Vieira V.V. (2024). *Bacillus lumedeiriae* sp. nov., a Gram-Positive, Spore-Forming Rod Isolated from a Pharmaceutical Facility Production Environment and Added to the MALDI Biotyper(®) Database. Microorganisms.

[83] Ruiz-García C., Béjar V., Martínez-Checa F., Llamas I., Quesada E. (2005). *Bacillus velezensis* sp. nov., a surfactant-producing bacterium isolated from the river Vélez in Málaga, southern Spain. Int. J. Syst. Evol. Microbiol..

[84] de Los Santos Villalobos S., Robles R.I., Parra Cota F.I., Larsen J., Lozano P., Tiedje J.M. (2019). *Bacillus cabrialesii* sp. nov., an endophytic plant growth promoting bacterium isolated from wheat (*Triticum turgidum* subsp. durum) in the Yaqui Valley, Mexico. Int. J. Syst. Evol. Microbiol..

[85] Dunlap C.A., Bowman M.J., Zeigler D.R. (2020). Promotion of *Bacillus subtilis* subsp. inaquosorum, *Bacillus subtilis* subsp. spizizenii and *Bacillus subtilis* subsp. stercoris to species status. Antonie Van Leeuwenhoek.

[86] Skerman V.B.D., McGOWAN V., Sneath P.H.A. (1980). Approved lists of bacterial names. Int. J. Syst. Bacteriol..

[87] Dunlap C.A., Kwon S.W., Rooney A.P., Kim S.J. (2015). *Bacillus paralicheniformis* sp. nov., isolated from fermented soybean paste. Int. J. Syst. Evol. Microbiol..

[88] Ma Q., Xiang X., Ma Y., Li G., Liu X., Jia B., Yang W., Yin H., Zhang B. (2024). Identification and Bioactivity Analysis of a Novel *Bacillus* Species, *B. maqinnsis* sp. nov. Bos-x6-28, Isolated from Feces of the Yak (*Bos grunniens*). Antibiotics.

