# Supplementary material for: Bacillus velezensis LW-66: A Broad-Spectrum Biocontrol Agent Against Apple Tree Canker and Other Plant Fungal Diseases
Source: Microorganisms. 2026 Apr 16;14(4):889. doi: 10.3390/microorganisms14040889 (PMC13119044; doi:10.3390/microorganisms14040889)
Supplement: Supplementary file 1 [file microorganisms-14-00889-s001.zip › microorganisms-4235794-New Supplementary.pdf]

Bacillus velezensis LW-66: A Broad-Spectrum Biocontrol Agent Against Apple Tree Canker and Other Plant Fungal Diseases

Dandan Liu [1,†], Wei Xiao [1,2,†] , Wenwen Li [1], Shengli Li [1,2], Juanli Cheng [1,2,*], Jinshui Lin [1,2,*]

[1] Shaanxi Key Laboratory of Research and Utilization of Resource Plants on the Loess Plateau, College of Life Sciences, Yan'an University, Yan'an 716000, China; 17829553016@163.com (D.L.); 18792388186@163.com (W.X.); 15129750408@163.com (W.L.); 18181017935@163.com (S.L.)

[2] State Key Laboratory for Crop Stress Resistance and High-Efficiency Production, Northwest A&F University, Yangling 712100, China

* Correspondence: chengjl@yau.edu.cn (J.C.), linjinshui@yau.edu.cn (J.L.)

† These authors contributed equally to this work.

Contents:

Supplementary Table S1

Supplementary Table S2

Supplementary Table S3

Supplementary Table S4

Supplementary Figure S1

Supplemental Materials

Table S1. The inhibitory rates of the strain LW-66 against *V. mali*.

Strain	colony diameter/mm	Disease prevention effect/%
CK	87.00±0.50	
LW-1	43.00±0.87	55.70±1.10
LW-2	67.33±1.04	24.89±1.32
LW-3	46.00±1.00	51.90±1.27
LW-4	69.83±1.15	21.73±1.46
LW-5	34.33±1.04	66.67±1.32
LW-6	26.83±0.76	76.16±0.97
LW-7	55.00±1.00	40.51±1.27
LW-8	63.83±1.15	29.32±1.46
LW-66	15.67±0.29	90.30±0.37
LW-10	85.50±0.50	1.90±0.63
LW-11	34.83±0.29	66.03±0.37
LW-12	76.17±0.76	13.71±0.97
LW-13	81.50±1.00	6.96±1.27
LW-14	20.50±1.50	84.18±1.90
LW-15	25.83±0.76	77.43±0.97

Table S2. Inhibitory effect of the strain LW-66 on the growth of five plant pathogenic fungi on PDA plates

Type	LW-66		extract		CK
	colony diameter/mm	Disease prevention effect/%	Colony diameter/mm	Disease prevention effect/%	
Valsa mali	15.67±0.29	90.19±0.37	14.50±1.32	91.68±1.69	86.17±0.29
Bipolaris sorokinianum	21.50±1.00	79.39±1.53	20.67±0.76	80.66±1.17	73.50±1.32
Exserohilum turcicum	17.67±0.76	84.11±1.26	19.50 ±1.32	81.10±2.17	68.83±0.58
Fusarium graminearum	32.67±0.58	69.67±0.71	28.50±5.77	74.80±7.09	89.33±0.29
Alternaria solani	24.00±1.00	78.23±1.36	22.50±0.50	80.27±0.68	81.50±1.00

Table S3. Inhibitory effects of volatile substances from strain LW-66 on the mycelial growth of five plant pathogenic fungi

Type	colony diameter/mm	CK	Disease prevention effect /%
Valsa mali	14.17±0.76	82.33±1.04	91.70±1.03
Bipolaris sorokinianum	40.67±1.26	70.33±1.89	47.59±2.02
Exserohilum turcicum	43.83±2.08	86.33±0.76	54.26±2.66
Alternaria solani	38.83±0.76	84.00±0.50	59.43±1.00
Fusarium graminearum	61.00±1.73	86.83±0.76	32.77±2.20

Table S4. Genomic characteristics of *Bacillus* sp. LW-66 phylogenetic tree members.

Strain	Authority	Synonyms	Percent G+C	No. proteins	Bioproject accession	Biosample accession	Assembly accession ID	dDDH	Diff. G+C Percent
B. bruguierae FJAT-51639	Liu et al.2025(75)	*Bacillus bruguierae*	36.0	4851	PRJNA1070399	SAMN39644991	GCA_037104725.1	10	10.63
B. rhizoplanae CIP 111899	Kämpfer et al., 2022(76)	*Bacillus rhizoplanae*	36.5	4267	PRJEB47722	SAMEA9995628	GCA_917563915.1	10.1	10.05
B. cereus ATCC 14579	Frankland et al, 1887(77)	*Bacillus cereus*	35.5	5316	PRJNA509739	SAMN10591533	GCA_006094295.1	10.1	11.16
B. cytotoxicus NVH 391-98	Guinebretière et al. 2013(78)	*Bacillus cytotoxicus*	36	3844	PRJNA13624	SAMN02598310	GCA_000017425.1	10.2	10.57
B. pinisoli GXH0341	Huang et al. 2023(79)	*Bacillus pinisoli*	37.0	3924	PRJNA786958	SAMN23765204	GCA_023036475.1	10	9.46
B. suaedaesalsae RD4P76	Xu et al. 2022(80)	*Bacillus suaedaesalsae*	36.0	3097	PRJNA700001	SAMN17817098	GCA_016890225.1	10	10.23
B. ectoiniformans DSM 28970	Zhu et al. 2016(81)	*Bacillus ectoiniformans*	41.0	3488	PRJNA695621	SAMN17620071	GCA_016908875.1	10.3	5.29
B. lumedeiriae B190/17	Costa et al. 2024(82)	*Bacillus lumedeiriae*	41.5	3231	PRJNA990794	SAMN36275113	GCA_044891285.1	10.3	4.87

B. velezensis NRRL B-41580	Ruiz-García et al. 2005(83)	*Bacillus velezensis*	46.5	3790	PRJNA299292	SAMN04196551	GCA_001461825.1	94.2	0.12
B. cabrialesii TE3	de Los Santos Villalobos et al. 2019(84)	*Bacillus cabrialesii*	44.0	3991	PRJNA504313	SAMN10390288	GCA_004124315.2	26.6	2.23
B. spizizenii TU-B-10	Dunlap et al. 2020(85)	*Bacillus spizizenii*	44	4296	PRJNA68561	SAMN02603352	GCA_000227465.1	26.6	2.62
B. subtilis ATCC 6051	Skerman et al. 1980(86)	*Bacillus subtilis*	43.5	4424	PRJNA553198	SAMN12236531	GCA_031316525.1	25.7	3.19
B. licheniformis ATCC 14580	Skerman et al., 1980(86)	*Bacillus licheniformis*	46.0	4187	PRJNA1037563	SAMN38186259	GCA_034478925.1	14.8	0.24
B. paralicheniformis KJ-16	Dunlap et al., 2015(87)	*Bacillus paralicheniformis*	46.0	4319	PRJNA282685	SAMN03573918	GCA_001042485.2	14.7	0.69
B. maqinnsis Bos-x6-28	Ma et al. 2024(88)	*Bacillus maqinnsis*	40.0	3306	PRJNA1128016	SAMN42024221	GCA_040500475.1	15.0	6.23
B. pumilus ATCC 7061	Skerman et al. 1980(86)	*Bacillus pumilus*	41.5	3900	PRJNA29785	SAMN02470256	GCA_000172815.1	12.6	4.78
Escherichia coli ATCC 11775	Skerman et al. 1980(86)	*Escherichia coli*	50.5	4723	PRJNA472652	SAMN10252913	GCA_003697165.2	9.4	4.2

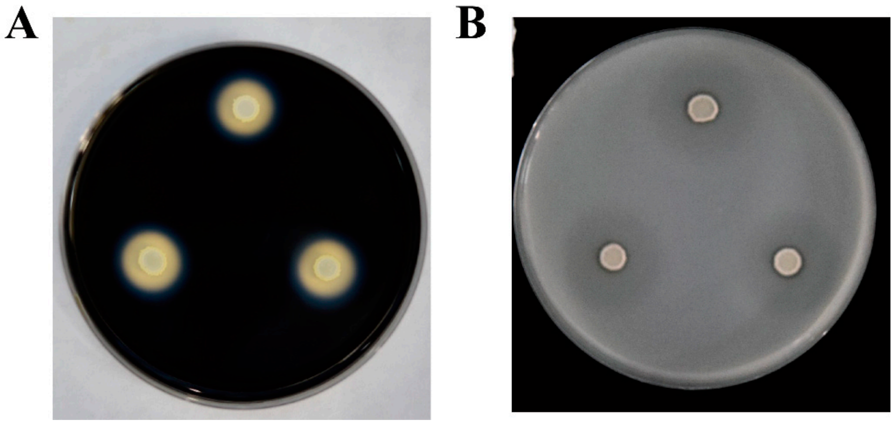

Figure S1. Analysis of the extracellular enzyme activities of strain LW-66. (A) Amylase activity of strain LW-66. (B) Protease activity of strain LW-66.

References

75. Liu, G.H.; Narsing Rao, M.P.; Shi, H.; Chen, Q.Q.; Quadri, S.R.; Li, W.J. Description of six novel species Bacillus bruguierae sp. nov., Bacillus kandeliae sp. nov., Bacillus yunxiaonensis sp. nov., Metabacillus rhizosphaerae sp. nov., Metabacillus sediminis sp. nov. and Psychrobacillus mangrovi sp. nov., isolated from mangrove ecosystem. *International journal of systematic and evolutionary microbiology* 2025, *75*, doi:10.1099/ijsem.0.006656.

76. Kämpfer, P.; Lipski, A.; McInroy, J.A.; Clermont, D.; Criscuolo, A.; Glaeser, S.P. Bacillus rhizoplanae sp. nov. from maize roots. *International journal of systematic and evolutionary microbiology* 2022, *72*, doi:10.1099/ijsem.0.005450.

77. Frankland, G.C.; Frankland, P.F. XI. Studies on some new micro-organisms obtained from air. *Philosophical Transactions of the Royal Society of London. (B.)* 1887, 257-287, doi:10.1098/rstb.1887.0011

78. Guinebretière, M.H.; Auger, S.; Galleron, N.; Contzen, M.; De Sarrau, B.; De Buyser, M.L.; Lamberet, G.; Fagerlund, A.; Granum, P.E.; Lereclus, D.; et al. Bacillus cytotoxicus sp. nov. is a novel thermotolerant species of the Bacillus cereus Group occasionally associated with food poisoning. *International journal of systematic and evolutionary microbiology* 2013, *63*, 31-40, doi:10.1099/ijs.0.030627-0.

79. Huang, Y.; Cai, H.; Qin, S.; Yang, L.; Zhou, Y.; Wu, J.; Chen, X.; Jiang, M.; Jiang, Y.; Ihsan, Y.N. Bacillus pinisoli sp. nov., Isolated from Soil of a Decayed Pine Tree. *Current microbiology* 2022, *80*, 55, doi:10.1007/s00284-022-03130-x.

80. Xu, L.; Huang, X.X.; Wang, H.T.; Tang, S.K.; Shen, B.; Sun, J.Q. Description and characterization of three endophytic Bacillaceae from the halophyte Suaeda salsa: Paenalkalicoccus suaedae gen. nov., sp. nov., Cytobacillus suaedae sp. nov., and Bacillus suaedae sp. nov. *International journal of systematic and evolutionary microbiology* 2022, *72*, doi:10.1099/ijsem.0.005337.

81. Zhu, D.; Zhang, P.; Niu, L.; Xie, C.; Li, P.; Sun, J.; Hang, F. Bacillus ectoiniformans sp. nov., a halotolerant bacterium isolated from deep-sea sediments. *International journal of systematic and evolutionary microbiology* 2016, *66*, 616-622, doi:10.1099/ijsem.0.000763.

82. Costa, L.V.D.; Ramos, J.N.; Albuquerque, L.S.; Miranda, R.; Valadão, T.B.; Veras, J.F.C.; Vieira, E.M.D.; Forsythe, S.; Brandão, M.L.L.; Vieira, V.V. Bacillus lumedeiriae sp. nov., a Gram-Positive, Spore-Forming Rod Isolated from a Pharmaceutical Facility Production Environment and Added to the MALDI Biotyper(®) Database. *Microorganisms* 2024, *12*, doi:10.3390/microorganisms12122507.

83. Ruiz-García, C.; Béjar, V.; Martínez-Checa, F.; Llamas, I.; Quesada, E. Bacillus velezensis sp. nov., a surfactant-producing bacterium isolated from the river Vélez in Málaga, southern Spain. *International journal of systematic and evolutionary microbiology* 2005, *55*, 191-195, doi:10.1099/ijs.0.63310-0.

84. de Los Santos Villalobos, S.; Robles, R.I.; Parra Cota, F.I.; Larsen, J.; Lozano, P.; Tiedje, J.M. Bacillus cabrialesii sp. nov., an endophytic plant growth promoting bacterium isolated from wheat (Triticum turgidum subsp. durum) in the Yaqui Valley, Mexico. *International journal of systematic and evolutionary microbiology* 2019, *69*, 3939-3945, doi:10.1099/ijsem.0.003711.

85. Dunlap, C.A.; Bowman, M.J.; Zeigler, D.R. Promotion of Bacillus subtilis subsp. inaquosorum, Bacillus subtilis subsp. spizizenii and Bacillus subtilis subsp. stercoris to species status. *Antonie van Leeuwenhoek* 2020, *113*, 1-12, doi:10.1007/s10482-019-01354-9.

86. Skerman, V.B.D.M., V.; Sneath, P.H.A. Approved lists of bacterial names. *International journal of systematic bacteriology* 1980, *30*, 225–420, doi:10.1099/00207713-30-1-225.

87. Dunlap, C.A.; Kwon, S.W.; Rooney, A.P.; Kim, S.J. Bacillus paralicheniformis sp. nov., isolated from fermented soybean paste. *International journal of systematic and evolutionary microbiology* 2015, *65*, 3487-3492, doi:10.1099/ijsem.0.000441.
88. Ma, Q.; Xiang, X.; Ma, Y.; Li, G.; Liu, X.; Jia, B.; Yang, W.; Yin, H.; Zhang, B. Identification and Bioactivity Analysis of a Novel Bacillus Species, B. maqinnsis sp. nov. Bos-x6-28, Isolated from Feces of the Yak (Bos grunniens). *Antibiotics (Basel, Switzerland)* 2024, *13*, doi:10.3390/antibiotics13121238.
